# The Emergence of 5-Year-Olds’ Behavioral Difficulties: Analyzing Risk and Protective Pathways in the United Kingdom and Germany

**DOI:** 10.3389/fpsyg.2021.769057

**Published:** 2022-01-05

**Authors:** Wei Huang, Sabine Weinert, Helen Wareham, James Law, Manja Attig, Jutta von Maurice, Hans-Günther Roßbach

**Affiliations:** ^1^Leibniz Institute for Educational Trajectories, Bamberg, Germany; ^2^Bamberg Graduate School of Social Sciences, University of Bamberg, Bamberg, Germany; ^3^Department of Developmental Psychology, University of Bamberg, Bamberg, Germany; ^4^School of Education, Communication and Language Sciences, Newcastle University, Newcastle upon Tyne, United Kingdom; ^5^Institute of Educational Science, University of Bamberg, Bamberg, Germany

**Keywords:** behavioral difficulties, protective pathways, risk pathways, parent–child interactions, negative discipline, vocabulary

## Abstract

This study aimed to advance our understanding of 5-year-olds’ behavioral difficulties by modeling and testing both mediational protective and risk pathways simultaneously. Drawing on two national samples from different Western European countries—the United Kingdom (13,053) and Germany (2,022), the proposed model considered observed sensitive parental interactive behaviors and tested child vocabulary as protective pathways connecting parental education with children’s behavioral outcomes; the risk pathways focused on negative parental disciplinary practices linking (low) parental education, parental distress, and children’s difficult temperament to children’s behavioral difficulties. Further, the tested model controlled for families’ income as well as children’s sex and formal child care attendance. Children with comparatively higher educated parents experienced more sensitive interactive behavior, had more advanced vocabulary, and exhibited fewer behavioral difficulties. Children with a comparatively higher level of difficult temperament or with parents who suffered from distress tended to experience more negative disciplinary behavior and exhibited more behavioral difficulties. Additionally, children’s vocabulary skills served as a mechanism mediating the association between parental education and children’s behavioral difficulties. Overall, we found similar patterns of results across the United Kingdom and Germany with both protective and risk pathways contributing simultaneously to children’s behavioral development. The findings suggest that promoting parents’ sensitive interactive behaviors, favorable disciplinary practices, and child’s vocabulary skills have potential for preventing early behavioral difficulties.

## Introduction

Early behavioral difficulties are a series of age-inappropriate behaviors such as inattention–hyperactivity or conduct problems. They often persist throughout elementary school and into adolescence and adulthood (Duncan and Magnuson, 2011). Previous research suggests that without intervention, early behavioral difficulties can become a crystallized pattern of behavior (e.g., Eron, 1994), increase academic problems such as school dropout, and influence later academic achievement (Rabiner et al., 2016). Children frequently do not “grow out of” early occurring behavioral problems naturally (Richman et al., 1982). Hence, a comprehensive identification of factors affecting early behavioral outcomes of overall typically developing children can facilitate proposing efficient preventive programs.

Evidence from longitudinal studies indicates that family’s socioeconomic status, parental behaviors, and child characteristics are important distal and proximal contributors to developmental pathways leading to behavioral difficulties (e.g., van Zeijl et al., 2007; Bates et al., 2014; Gard et al., 2020). However, previous studies focused mainly on dysfunctional pathways (i.e., risk factors as mediating pathways) to behavioral difficulties (e.g., Conger et al., 2000; Gard et al., 2020). Protective pathways (i.e., protective factors as mediating pathways) that may hinder the development of behavioral difficulties are still understudied and cannot be viewed exclusively as an absence of risk factors. Which pathways are more pronounced when risk and protective factors are considered simultaneously remains an open question, because both channels are likely to occur together in this developmental process (Gore and Eckenrode, 1996).

Drawing on theoretical assumptions and empirical evidence, the present study proposes and simultaneously tests a complex model of protective and risk pathways to children’s behavioral difficulties. In particular, given the importance of parenting behavior in socialization processes in early childhood (Ainsworth, 1979), this study investigated the effects of two different forms of parenting behaviors as mediating protective and risk pathways—that is, how sensitive parent–child interactions and negative disciplinary practices differentially affect children’s behavioral outcomes. Our models also considered central characteristics that have been shown to influence both parenting behaviors and children’s behavioral outcomes: parental education, parental psychological distress, and child’s difficult temperament (Deater-Deckard, 2004; McLearn et al., 2006; Bates et al., 2014; Weinert et al., 2017; Hoff and Laursen, 2019). Further, we investigated whether the effect of parental interactive behaviors is mediated by advanced early vocabulary skills which may additionally serve as a protective pathway influencing children’s behavioral outcomes, because language is a key developmental asset facilitating cognitive and behavioral development (Vygotsky, 1978; Bruner, 1985). Finally, we tested the generalizability of the studied mechanisms by sequentially running the model using two national representative data.

### Protective Pathways

Parental education has been widely studied as an indicator of family background predicting children’s behavioral outcomes (e.g., Hoff et al., 2002; Hoff and Laursen, 2019). Children with higher educated parents exhibited more competent behavior, whereas children with lower educated parents displayed significantly poorer social-emotional behavior than children with higher educated parents (Halle et al., 2009). One way in which parental education is assumed to affect children’s behavior is by shaping parent–child interactions and parental disciplinary practices—that is, what parents do in discipline encounters (Weinert et al., 2017; Hoff and Laursen, 2019). Higher educated parents generally have more resources that matter for children’s development (Coleman, 1988) such as books, toys, and access to child care programs. Furthermore, they generally acknowledge the importance of enriched learning environments, making them more likely to provide children with environments that foster their development such as supportive (i.e., sensitive, warm) interactions (e.g., Weinert et al., 2017). Compared to other important components of family background, i.e., family income and occupation (which might fluctuate over time), parental education is relatively stable and easy to measure. In addition, parental education has been found to influence later income and occupation (Cohn and Addison, 1998) and to explain the greatest share of the variance in child outcomes (Hoff et al., 2002). Thus, for this study, we chose parental education as an indicator of family background contributing to the pathways leading to differences in children’s behavioral outcomes.

#### Sensitive Parent–Child Interactions

Attachment theory indicates that parental sensitivity and warmth in responding to infant behavior can establish a secure parent–child attachment (Ainsworth, 1979). In particular, such sensitive interactive behaviors are more effective than other parenting behaviors (such as negative discipline) in providing a secure base for developing autonomy and self-regulation in toddlerhood (Ainsworth et al., 1974; Grusec and Goodnow, 1994; Ispa et al., 2017). Hence, these kinds of sensitive parent–child interactions in early childhood may serve as a protective factor against the development of behavioral difficulties from an early age. This is empirically supported by study results on the effects of sensitive parent-child interactions on children’s behavioral outcomes (e.g., Sanders et al., 2000; Gardner et al., 2007; Barnett et al., 2012; Huang et al., forthcoming). For example, drawing on a low-income sample, Gardner et al. (2007) found that proactive parent–child interactions (including the use of constructive activities, positive disciplinary strategies, and praise) were associated negatively with children’s destructive behavior over and above the effects of negative disciplinary practices. Moreover, using a national sample from Germany, a recent study found that supportive parent–child interactions at 26 months was associated negatively with peer problems at 38 months (Huang et al., forthcoming). To enhance our understanding of this protective mechanism, we examined a protective pathway linking parental education and children’s behavioral difficulties *via* an effect of sensitive parental interactive behavior on children’s behavioral outcomes (Pathway a).

#### Subsequent Mechanism—Language

Apart from the positive effects of protective factors *per se*, some protective factors may also impact on the later emergence of other protective mechanisms (Gore and Eckenrode, 1996). Sensitive parental interactive behaviors have also been suggested to impact children’s language development by appropriately responding to children (e.g., Barnett et al., 2012; Nozadi et al., 2013; Bornstein et al., 2020). Furthermore, sensitive parents are also more likely to provide children with a verbally stimulating learning environment that facilitates the acquisition of advanced language skills (Huang et al., forthcoming). Subsequently, higher language skills provide children with essential means of communication and self-regulation of their social behavior (Vygotsky, 1978; Bruner, 1985). This facilitates their efforts to interpret social exchange, to communicate better with peers, and to show more cooperative behavior (e.g., Vygotsky, 1978; Rose et al., 2018).

In particular, receptive and expressive vocabulary skills in early childhood such as understanding words and putting ideas into words are associated with later behavioral outcomes (e.g., Menting et al., 2011; Girard et al., 2016; Rose et al., 2018; Petersen and LeBeau, 2021). Advanced receptive language skills at age 3 significantly predict the development of cooperative behavior between ages 3 and 7 (Rose et al., 2018), and better receptive language at age 4 predicts decreased externalizing behavioral problems by 6 years (Petersen and LeBeau, 2021). Conversely, both limited receptive and expressive language skills prevent children from expressing themselves well, leading to potentially increased peer rejection and to a higher risk of exhibiting behavioral difficulties such as aggressive or inattention–hyperactive behaviors (e.g., Menting et al., 2011; Girard et al., 2016).

In this regard, more advanced vocabulary skills are probably a protective factor, whereas limited vocabulary skills may become a risk factor for behavioral development. Therefore, we specified two more pathways linking parental education and children’s behavioral difficulties: one directly *via* an effect of children’s vocabulary on behavioral outcomes (Pathway b) and another indirectly *via* an effect of parental sensitive interactions on children’s behavioral difficulties mediated by child vocabulary (Pathway c).

### Risk Pathways

When considering how risk factors influence children’s behavioral development, studies draw mainly on the family stress model (FSM) hypothesizing that stressors lead to children’s adverse behavioral outcomes indirectly through family processes such as negative disciplinary parenting practices (Conger et al., 2000). Empirical evidence suggests that parents suffering from higher levels of stress are more likely to show less engagement and consistency in interactions with their children (McLearn et al., 2006) and tend to exhibit more coercive behaviors and negative emotional expressions in disciplinary encounters—that is, to use harsher and more inconsistent strategies to discipline their children (Deater-Deckard, 2004). Subsequently, by experiencing a series of negative disciplinary encounters, children are at risk for behavioral difficulties at a very early age (e.g., van Zeijl et al., 2007; Choe et al., 2013; Gard et al., 2020). In addition, children’s characteristics such as temperament also play an important role in both parenting behavior and children’s behavioral outcomes. In particular, children with an early difficult temperament are not only more likely to develop behavior problems (van Zeijl et al., 2007), but also less likely to comply with parents’ socialization efforts, and they tend to elicit harsher and more inconsistent discipline from their parents (Bates et al., 2014), leading to another pathway to children’s early behavioral difficulties. Given such evidence, we specified the first two risk pathways leading from parental psychological distress as well as from child’s difficult temperament *via* parents’ negative disciplinary practices to children’s behavioral difficulties (Pathways d and e).

Although FSM emphasizes the prominent role of economic pressure, parental education might also play an important role in their disciplinary practices. Parents with comparatively lower education might have fewer resources and capacities to help them cope with stressors, and they are more likely to use negative strategies in discipline encounters than higher educated parents (Ryan et al., 2016). Thus, we hypothesized an additional risk pathway from parental education *via* negative disciplinary practices to children’s behavioral difficulties (Pathway f).

### Co-occurrence of Protective and Risk Pathways

Despite existing evidence on mediating pathways related to children’s behavioral difficulties, it is important to consider whether protective pathways make an independent contribution to children’s behavioral outcomes over and above the effects of risk pathways and vice versa. So far, empirical findings on co-occurrences of these factors have been mixed (e.g., Gardner et al., 2003; McKee et al., 2007; Totsika et al., 2019). Gardner et al. (2003) found that protective factors (i.e., sensitive mother–child interactions) correlated uniquely with fewer conduct problems at age 4 independent of other risk factors including low SES, children’s initial conduct problems and hyperactivity, maternal depression, and negative parenting behavior at age 3. In contrast, a recent study by Totsika et al. (2019) found that in risk circumstances (i.e., children with intellectual disability, experience of poverty, and parental psychological distress at 9 months), the effect of the modeled protective pathway (indicated by positive parent–child relationship at ages 3 and 5) on children’s behavioral difficulties at age 7 disappeared when the negative pathway (indicated by negative parenting behavior at ages 3 and 5) was included in the analysis. Conversely, the negative pathway maintained a robust effect on children’s behavioral difficulties regardless of the presence of the protective pathway.

This inconsistent evidence makes it hard to build up a comprehensive picture of what happens when multiple risk and protective factors appear together. Hence, this study studied multiple protective and risk pathways linking children’s early behavioral outcomes simultaneously with the aim of establishing more complex understanding of the mechanisms involved. In particular, we examined whether the predicted pattern of mechanisms holds across different birth cohort studies conducted in different Western European countries (the United Kingdom and Germany) at different time points (and thus also across comparable but slightly different instruments and operationalizations of the central constructs).

### The Present Study

Our primary goal was to advance our understanding of the underlying mediating protective and risk pathways connected to children’s behavioral difficulties at age 5. However, according to Spanos (1986), theoretical assumptions cannot be unambiguously translated into an empirically testable model, due to the fact that not all constructs and relations are directly observable and may be measured differently. In other words, as multiple factors (operationalized in given ways) can constitute these protective or risk pathways, it is not possible to include all of these factors in our model due to restricted data. To address these issues, we applied the probabilistic reduction approach to specify our empirical model, which captures the systematic nature of the observed data (Spanos, 1986; Kaplan, 2009). That is, guided by theoretical assumptions, we chose central influential and observable factors from two different data sets and modeled their relations within a structural equation model to address our research questions empirically. The protective pathways consisted of parental education through sensitive parent–child interactions and children’s vocabulary skills to children’s behavioral outcomes. The risk pathways to behavioral difficulties focused on parental education, parental psychological distress, and the child’s difficult temperament through parents’ negative disciplinary practices. In relation to protective and risk pathways in early childhood, it is important to consider some additional characteristics that reflect diversity in view of their association with early behavioral difficulties. Thus, based on previous evidence, all analyses controlled for the child’s sex and formal child care attendance (under 36 months) that might influence children’s language and behavioral development (Barnett and Scaramella, 2013; Linberg et al., 2019a), as well as family net income that might affect negative disciplinary practices and children’s behavioral outcomes (Ryan et al., 2016).

Furthermore, we aimed to test for the generalizability of results across Western European countries by using two large longitudinal national samples from the United Kingdom and Germany. On the one hand, these two cultures emphasize individualism and independence and thus provide parents with similar implicit or explicit models for childrearing such as comparable behaviors that parents appreciate and emphasize (Bornstein and Cheah, 2006). On the other hand, they had contrasting welfare systems at the time of data collection: a liberal market economy in the United Kingdom (2000–2001) vs. a more conservative welfare state in Germany (2012–2013). The United Kingdom had less welfare state protection in terms of, e.g., paid parental leave (14 weeks) to support working parents (Daly and Scheiwe, 2010). Germany, in contrast, legally recognizes the family as a key societal institution (Fagnani, 2007) and has a series of regulations supporting families such as universal provisions for children and longer paid parental leave (12–14 months). These welfare resources are intended to promote parental health and behavior and, in turn, child development (e.g., Waldfogel, 2006). Drawing on these two perspectives, similar results will allow a generalization of the microlevel mechanisms across different Western European countries, albeit minor differences in the assessments and operationalizations employed, whereas different results could reflect effects of the social and educational systems and/or policy differences (and/or assessment differences). We explored the following three research questions:

(1) Do the specified protective and risk pathways account for early behavioral outcomes when being considered simultaneously?
  (a) In particular, we expect parents’ sensitive interactive behavior to partially mediate positive effects of parental education on children’s behavioral outcomes.
  (b) We expect child vocabulary to exert a direct effect on behavioral outcomes and to find an indirect link between parental education *via* sensitive interactive behaviors and language to children’s behavioral outcomes.
  (c) With respect to risk pathways, we expect parental education, psychological distress, and children’s difficult temperament to be risk factors related to children’s behavioral difficulties partially *via* negative disciplinary practices.
(2) Are the investigated pathways generalizable across countries?

As a robustness check we also test whether considering protective and risk pathways separately leads to the comparable results.

## Materials and Methods

### Participants

#### The Millennium Cohort Study

The Millennium Cohort Study (MCS) is an ongoing, multidisciplinary cohort study that began in 2000–2001. The MCS drew a representative sample of 18,552 families from across the United Kingdom in the first wave. Applying sample design weights permits statements for the whole United Kingdom. The MCS collects a diverse range of data from children, their siblings, and parents (Hansen et al., 2010). There have been seven waves to date (ranging from 9 months to 17 years). This study used data from Waves 1 (9 months), 2 (3 years), and 3 (5 years). Twins and the refreshment sample (in Wave 2, due to information that is not available in this sample) were excluded from the analyses. Hence, the current study included information on 13,053 children who participated in the third panel wave (49% female, age at Time 1: *M* = 9.20 months, *SD* = 0.51). In Wave 1, parents provided information on family’s demographic and children’s characteristics. Parents reported their highest vocational and academic qualifications under the National Vocational Qualification (NVQ) framework: low = 40.27%; middle = 16.75%; high = 42.98% (details see below) and their monthly family net equivalent income (*M* = 1,334.19 pounds, *SD* = 868.38; for details see below). In Wave 2, children were presented with a standardized vocabulary test.

#### The National Educational Panel Study

The ongoing German National Educational Panel Study (NEPS; Blossfeld and Roßbach, 2019; doi: 10.5157/NEPS:SC1:6.0.0) is a large-scale national longitudinal study addressing educational processes and trajectories. The current study used the newborn cohort study of the NEPS containing a representatively drawn sample of infants born across Germany from February to June 2012 (Aßmann et al., 2015). In Wave 1 in 2012–2013, a total of 3,431 families agreed to take part. The data set offers information on the target children and their families. In the children’s first 5 years, six measurement waves were conducted when children were around 7, 14, 26, 38, 50, and 60 months old. The current study included 2,022 children who participated in Wave 6 (50% female, age at Time 1: *M* = 7.10 months, *SD* = 0.74). Parents provided demographic information and reported on children’s characteristics at each measurement point. In Wave 1, parents reported their years of education (*M* = 15.83, *SD* = 2.24), and their monthly family net equivalent income (*M* = 1,723.86 Euro, *SD* = 868.53; for details see below). At 38 months, children’s vocabulary skills were assessed by a standardized test.

The current study only conducted secondary data analysis on both data sets and thus there was no need for Institutional Review Board approval.

### Measures

#### Children’s Behavioral Difficulties

In both data sets, parents rated their children’s behavior on three subscales from the Strengths and Difficulties Questionnaire (SDQ; Goodman, 1997)—Conduct Problems (e.g., often fights with other children), Hyperactivity (e.g., easily distracted, concentration wanders), and Peer Relationship Problems (e.g., picked on or bullied by other children)—using a 3-point scale ranging from 0 (*not true*) to 2 (*certainly true*). Each subscale contains five items and measures specific child behaviors. A confirmatory factor analysis (CFA) demonstrated good fits for these subscales within a second-order model containing three factors (MCS: χ^2^ = 2704.908, *df* = 87, *p* < 0.001, CFI = 0.938, RMSEA = 0.049, SRMR = 0.056, factor loadings = 0.48–0.83; NEPS: χ^2^ = 194.240, *df* = 87, *p* < 0.001, CFI = 0.954, RMSEA = 0.025, SRMR = 0.065, factor loadings = 0.41–0.86) compared to a first-order model containing one factor [MCS: Δχ^2^ = 2612.674 (3), *df* = 90, *p* < 0.001, CFI = 0.875, RMSEA = 0.068, SRMR = 0.083, factor loadings = 0.30–0.80; NEPS: Δχ^2^ = 223.155 (3), *df* = 90, *p* < 0.001, CFI = 0.860, RMSEA = 0.042, SRMR = 0.099, factor loadings = 0.30–0.83]. Thus, the following analyses used the factor scores extracted from the second-order model to indicate children’s behavioral difficulties. Higher scores suggested a higher level of behavioral difficulties.

#### Parental Education

Parent-reported education according to the country-specific education system was recoded into the Comparative Analysis of Social Mobility in Industrial Nations-Classification (CASMIN-Classification; Brauns et al., 2003). This consists of nine educational categories tracking both academic and vocationally oriented education that reflect institutional differences in national education and training systems, thereby facilitating the comparability of educational attainments in the United Kingdom and Germany. Because CASMIN indicators were already available in the NEPS data set, we recoded education attainment in the MCS into the CASMIN classification. Due to low numbers in certain categories, the CASMIN indicators in both cohorts were condensed into three groups: low, middle, and high (for details, see Supplementary Table 1).

#### Parental Psychological Distress

For MCS, parents reported their distress on nine items from the modified version of the Rutter Malaise Inventory (Rutter et al., 1970) when their children were about 9 months old. This binary inventory measures whether a parent generally suffers from an emotional disturbance (e.g., feel miserable) and experiences physical symptoms (e.g., heart races like mad). Cronbach’s alpha was 0.72. In NEPS, when children were about 7 months old, parents rated three items on their experienced depressive feelings or stress on a 5-point scale ranging from 1 (*never*) to 5 (*always*). An example is, “How often did you feel depressed and sad?” Cronbach’s alpha was 0.65.

#### Difficult Temperament (Child)

In MCS, parents reported children’s difficult temperament at 9 months using the 3-item Intensity subscale from the Carey Infant Temperament Scale (Carey and McDevitt, 1978) on a 5-point scales ranging from 1 (*almost never*) to 5 (*almost always*). Similarly, at 7 months, parents in NEPS reported their children’s behavior on the 3-item Negative Affectivity subscale of the Infant Behavior Questionnaire—Revised (IBQ–R; Gartstein and Rothbart, 2003) on 7-point scales ranging from 0 (*never*) to 6 (*always*). Both subscales indicate whether children exhibited challenging behavior over the past weeks (e.g., child becomes upset when not getting what he wants in the MCS; child gets angry when denied something special in NEPS). Cronbach’s alphas of both measurements were 0.54 (MCS) and 0.51 (NEPS).

#### Negative Disciplinary Practices

In MCS, harsh discipline was assessed with six items from the Conflict Tactics Scale (Straus and Hamby, 1997). When children were aged about 38 months, parents reported how often they have applied harsh disciplinary strategies (e.g., ignore child, shout at child) on 5-point scales ranging from 1 (*never*) to 5 (*always*). For NEPS, self-reported inconsistent disciplinary practices were derived from the Alabama Parenting Questionnaire (APQ; Shelton et al., 1996). Parents reported on four items how often they utilized inconsistent disciplinary practices (e.g., it’s hard for you to be resolute in your parenting) on a 5-point scale ranging from 1 (*never*) to 5 (*very often*) when children were aged 4 years. Cronbach’s alphas of both subscales were 0.70 (MCS) and 0.67 (NEPS).

#### Sensitive Parent–Child Interactions

In MCS, interviewers used selected items from the Home Observation for Measurement of the Environment scale (HOME; Bradley and Caldwell, 1984) to evaluate the physical environment, responsivity of the parent, and arrangement of the environment during home visits by indicating a yes (= 1) or no (= 2) on whether they observed certain parent--child interactions^1^. This study included six items capturing observed sensitive interactive behaviors at 38 months (e.g., the interviewed parent praised the child spontaneously). Cronbach’s alpha of this measurement was 0.60. The sensitive parent–child interactions in MCS were rated on binary scales (1 = yes, 2 = no), and about 90% parents were rated as showing sensitive interactions (= 1) on each of the six items (situations). We recoded this 90% as 1 (*providing prominent sensitive interactions*) and the remaining 10% as 0 (*providing non-prominent sensitive interactions*).

In NEPS, a measure adapted from the NICHD (National Institute of Child Health and Human Development) Study of Early Child Care and Youth Development (SECCYD; NICHD Early Child Care Research Network, 1999) was used as a basis to code the videotaped parent–child interactions in a semistructured play situation in the family homes at age 26 months. Parents were asked to play with their child as naturally as possible for 10 min using a standardized set of toys (for details, see Linberg et al., 2019b). Trained coders rated each 10-min videotape on qualitatively defined 5-point scales adapted from the rating instrument of the NICHD-SECCYD Study ranging from 1 (*not at all characteristic*) to 5 (*very characteristic*). Interrater reliability was good (weighted percentage between 92 and 94%). For the current study, five coded dimensions (items) of parent–child interactions were used: sensitivity to non-distress, positive regard for the child, emotionality, general stimulation, and language stimulation. Cronbach’s alpha was 0.80. For the following analyses, we first calculated the mean values of these five dimensions and then recoded the upper 90% of parents as 1 (*providing prominent sensitive interactions*) and the remaining 10% as 0 (*providing non-prominent sensitive interactions*).

#### Vocabulary Skills (Child)

In both cohorts, child vocabulary was assessed when children averaged 38 months old (MCS: *SD* = 2.50 months; NEPS: *SD* = 1.10 months). In MCS, children’s expressive vocabulary was assessed using the Naming Vocabulary subtest from the British Ability Scales Second Edition (BAS II; Elliott et al., 1996). The scale comprises a stimulus booklet presenting a total of 36 colorful pictures that the child is asked to name (e.g., picture of a shoe). Starting and stopping points differ depending on the child’s age and performance: the better they do, the more items they are given. The test is stopped at any point when the child has made five consecutive errors.

NEPS used the German version of the Peabody Picture Vocabulary Test—4 (PPVT—4; Dunn and Dunn, 2007) to measure children’s receptive vocabulary. The child was asked to select the one out of four pictures that best matched the meaning of the given word. Similar to the Naming Vocabulary subtest, starting and stopping points depended on the child’s age and performance. A maximum of 19 sets with 12 items each were administered, and the test was terminated when the child gave more than seven wrong answers in one set.

The standardized ability score (adjusted for item difficulty and age) from the Naming Vocabulary and an age-adjusted sum score of PPVT—4 were used as continuous variables indicating children’s vocabulary skills. Please note that although measurements of vocabulary skills differ across these two cohorts (i.e., expressive vocabulary vs. receptive vocabulary), both types of vocabulary skills are highly interrelated (see, e.g., Vatalaro et al., 2018).

#### Covariates

In MCS, the available weekly family net equivalent income was multiplied by 52 and divided by 12 to provide the monthly family net equivalent income. In NEPS, this was calculated using the OECD-modified equivalence scale (Hagenaars et al., 1994) that assigns a weight of 1.0 to the household head, 0.5 to each additional person older than 14 years, and 0.3 to each child under 14 years. Further, we log-transformed the net equivalent income from both data sets to reduce its skewness.

At 9 months (MCS) or 7 months (NEPS), parents in both data sets reported their child’s sex. Information on formal child care attendance under 36 months was gathered when children were around 9 and 36 months old in MCS and around 7, 14, 26, and 38 months old in NEPS. Formal child care was defined as childminder, day nursery, and playgroup in MCS, and center-based child care and childminder in NEPS.

### Analysis Strategy

Due to the non-normal distribution of the variables under study, a maximum likelihood estimator with robust standard errors (MLR) was used within structural equation modeling in M*plus* 8.3 (Muthén and Muthén, 2017). Full-information maximum likelihood (FIML) was used to handle missing data. All analyses included survey weights to account for the stratified cluster sample design of the studies and attrition bias due to non-response across surveys. Prior to the SEM analysis, three parcels were separately created by calculating the mean value of the items for parental psychological distress, difficult temperament, and negative disciplinary practices (Little et al., 2002; Matsunaga, 2008). Using parcels was preferred because it requires fewer parameters and is more parsimonious compared to item-level data (e.g., Little et al., 2002, 2013).

We ran the main model simultaneously examining pathways a to e. Both children’s vocabulary skills and behavioral difficulties were regressed on all covariates; sensitive parent–child interactions were regressed on family equivalent income; negative disciplinary practices were regressed on family equivalent income and formal childcare attendance. As robustness check, we ran two additional models to test whether protective and risk pathways revealed independent effects on children’s behavioral difficulties. Model A1 modeled only the protective pathways a, b, and c with sensitive parental interactive behaviors and vocabulary skills linking parental education and children behavioral outcomes. In Model A2, the risk pathways d, e, and f included parental education, parental psychological distress, and child’s temperament as risk factors linking to child behavioral difficulties *via* negative disciplinary practices.

In order to test the generalizability of the studied mechanisms, we sequentially ran the models using the MCS and NEPS data sets. For working model see Supplementary Figure 1.

## Results

### Descriptive Statistics

The descriptive statistics and bivariate correlations between all study variables in MCS and NEPS, respectively, are presented in Tables 1, 2. Unless noted otherwise, the significance level was set at *p* < 0.001 for all significant effects in the correlation analyses. In both countries, children with higher educated parents were more likely to exhibit fewer behavioral difficulties at age 5 (MCS: *r* = –0.25; NEPS: *r* = –0.23), to experience comparatively higher levels of supportive parent–child interactions at age 3 and 26 months, respectively (MCS: *r* = 0.23; NEPS: *r* = 0.34), and to show advanced vocabulary skills at age 3 (MCS: *r* = 0.40; NEPS: *r* = 0.28). Children who were reported to have a comparatively higher level of difficult temperament by their parents or whose parents suffered higher levels of psychological distress during the first year of their children’s lives were more likely to exhibit behavioral difficulties (MCS: *r* = 0.24 and 0.10; NEPS: *r* = 0.12 and 0.09). Furthermore, children who experienced a higher level of negative discipline tended to have more behavioral difficulties (MCS: *r* = 0.23; NEPS: *r* = 0.19), whereas those who experienced higher levels of sensitive parent–child interactions or had advanced vocabulary skills were less likely to exhibit behavioral difficulties (MCS: *r* = –0.13 and –0.23; NEPS: *r* = –0.10 and –0.12).

**TABLE 1:** Descriptive statistics of study variables (MCS: *N* = 13,053; NEPS: *N* = 2,022).

	MCS					NEPS				
*Variable*	*M*	*SD*	Min	Max	Range	*M*	*SD*	Min	Max	Range
**Outcomes** (5 years)										
Behavioral difficulties (Mean value of three subscales)	0.40	0.28	0.00	1.93	0–2	0.38	0.22	0.00	1.60	0–2
**Predictors** (9/7 months)										
Parental education	2.03	0.91	1.00	3.00	1–3	2.50	0.60	1.00	3.00	1–3
Parental psychological distress	0.18	0.19	0.00	1.00	0–1	1.75	0.60	1.00	4.33	1–5
Difficult temperament (Child)	2.76	0.88	1.00	5.00	1–5	3.67	1.20	0.00	6.00	0–6
**Mediators**										
Negative disciplinary practices (3/4 years)	2.85	0.72	1.00	5.00	1–5	2.54	0.63	1.00	5.00	1–5
Sensitive parent–child interactions (3 years/26 months)	0.91	0.17	0.00	1.00	0–1	3.50	0.59	1.60	5.00	1–5
Sensitive parent–child interactions (Dichotomized)	0.90	0.30	0.00	1.00	0–1	0.91	0.29	0.00	1.00	0–1
Vocabulary skills (Child; 3 years; Sum scores/Age-adjusted scores)	73.77	17.69	10.00	141.00	10–141	48.83	27.86	0.00	121.00	0–228
**Control variables**										
Family net equivalent income (£/€)	1,334.19	868.38	62.62	5,558.67	62.62–5,558.67	1,758.71	898.66	270.56	14,285.71	270.56–14,285.71
Child’s sex (Girls = 1)	0.49	0.50	0.00	1.00	0–1	0.50	0.50	0.00	1.00	0–1
Formal childcare attendance (Under 36 months; in months)	3.36	7.76	0.00	36.00	0–36	15.31	8.59	0.00	30.00	0–36

**TABLE 2:** Bivariate correlations between study variables: MCS (above diagonal) and NEPS (below diagonal).

		1	2	3	4	5	6	7	8	9	10
1.	Behavioral difficulties (5 years)	—	–0.25***	0.24***	0.10***	0.23***	–0.13***	–0.23***	–0.26***	–0.15***	–0.04***
2.	Parental education (9/7 months)	–0.23***	—	–0.11***	0.06***	0.03**	0.14***	0.27***	0.53***	0.01	0.14***
3.	Parental psychological distress (9 months)	0.12***	–0.06**	—	0.19***	0.12***	–0.08***	–0.07***	–0.16***	–0.02*	–0.01
4.	Difficult temperament (Child; 9/7 months)	0.09***	0.00	0.21***	—	0.17***	–0.02*	–0.01	0.00	–0.05***	0.02*
5.	Negative disciplinary practices (3 years)	0.19***	–0.07**	0.16***	0.14***	—	–0.01	0.02*	0.03***	–0.10***	0.04***
6.	Sensitive parent–child interactions (26 months/3 years)	–0.09***	0.16***	–0.05	–0.05	–0.03	—	0.17***	0.18***	0.03**	0.05***
7.	Vocabulary skills (Child; 3 years)	–0.12***	0.17***	0.00	–0.01	0.02	0.07*	—	0.31***	0.11***	0.06***
8.	Family net equivalent income (9/7 months)	–0.16***	0.43***	–0.14***	0.06*	–0.07**	0.16***	0.09**	—	0.00	0.13***
9.	Child’s sex (9/7 months; Girls = 1)	–0.15***	0.03	0.01	–0.02	–0.02	0.00	0.03	–0.00	—	–0.00
10.	Formal childcare attendance (Under 36 months; in months)	–0.05*	0.23***	0.00	–0.01	0.01	0.03	0.06*	–0.02	–0.02	—

*p < 0.05, **p < 0.01, ***p < 0.001.

### Main Models

**FIGURE 1:**
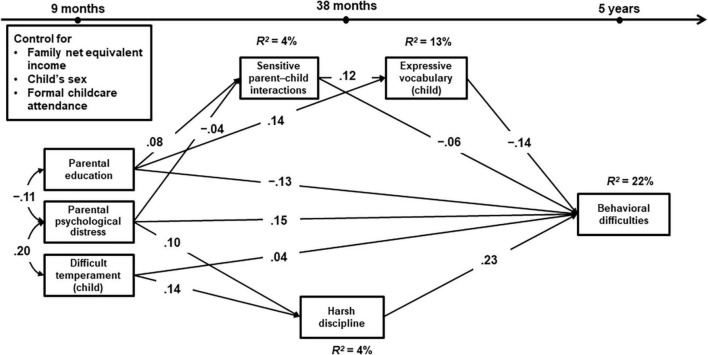
Standardized estimates of the conditional Model A1 using MCS in United Kingdom. All coefficients are significant at the *p* < 0.05 level. *N* = 13,053, χ^2^ = 100.635, *df* = 10, *p* < 0.001, CFI = 0.981, RMSEA = 0.026, and SRMR = 0.013.

**FIGURE 2:**
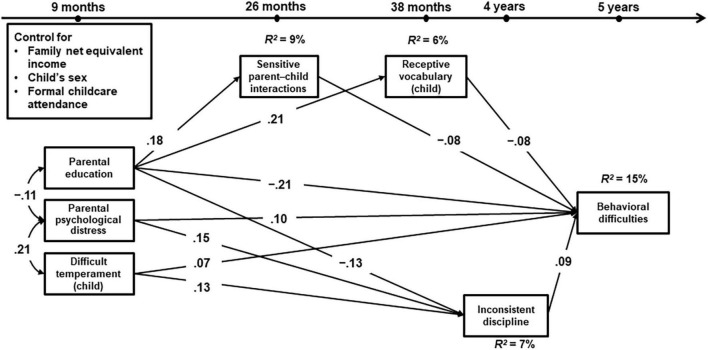
Standardized estimates of the conditional Model A1 using NEPS in Germany. All coefficients are significant at the *p* < 0.05 level. *N* = 2,022, χ^2^ = 12.133, *df* = 10, *p* = 0.35, CFI = 0.995, RMSEA = 0.007, and SRMR = 0.016.

We found very similar results in the United Kingdom and Germany. The model fit indices indicated that the models fit both data well (MCS: χ^2^ = 100.635, *df* = 10, *p* < 0.001, CFI = 0.981, RMSEA = 0.026, SRMR = 0.013; NEPS: χ^2^ = 11.133, *df* = 10, *p* = 0.35, CFI = 0.995, RMSEA = 0.007, SRMR = 0.016). The results of the structural equation models are presented in Table 3. Table 4 gives an overview of the total and indirect effects (via different pathways) to children’s behavioral difficulties. Figures 1, 2 include all significant paths with standardized estimates (*p* < 0.05).

**TABLE 3:** Results from structural equation modeling for MCS and NEPS.

	MCS			NEPS		
	*B (SE)*	β (*SE*)	*p*	*B (SE)*	β (*SE*)	*p*
**Behavioral difficulties →**						
Parental education	–0.08 (0.01)	–0.13 (0.01)	**0.00**	–0.14 (0.03)	–0.21 (0.04)	**0.00**
Parental psychological distress	0.42 (0.03)	0.15 (0.01)	**0.00**	0.08 (0.03)	0.12 (0.04)	**0.01**
Difficult temperament	0.03 (0.01)	0.04 (0.01)	**0.00**	0.03 (0.01)	0.07 (0.04)	**0.05**
Negative disciplinary practices	0.17 (0.01)	0.23 (0.01)	**0.00**	0.06 (0.03)	0.09 (0.04)	**0.03**
Sensitive parent–child interactions	–0.03 (0.01)	–0.06 (0.01)	**0.00**	–0.11 (0.06)	–0.08 (0.04)	**0.05**
Vocabulary skills	–0.07 (0.01)	–0.14 (0.01)	**0.00**	–0.04 (0.02)	–0.08 (0.04)	**0.03**
**Negative disciplinary practices →**						
Parental education	0.00 (0.01)	0.00 (0.01)	0.86	–0.13 (0.05)	–0.13 (0.04)	**0.01**
Parental psychological distress	0.39 (0.04)	0.10 (0.01)	**0.00**	0.17 (0.04)	0.15 (0.04)	**0.00**
Difficult temperament (Child)	0.11 (0.01)	0.14 (0.01)	**0.00**	0.07 (0.02)	0.13 (0.04)	**0.00**
**Sensitive parent–child interactions →**						
Parental education	0.13 (0.01)	0.11 (0.01)	**0.00**	0.09 (0.03)	0.18 (0.06)	**0.00**
Parental psychological distress	–0.24 (0.07)	–0.04 (0.01)	**0.00**	0.00 (0.02)	0.01 (0.04)	0.88
**Vocabulary skills →**						
Parental education	0.13 (0.01)	0.12 (0.01)	**0.00**	0.31 (0.07)	0.21 (0.04)	**0.00**
Sensitive parent–child interactions	0.20 (0.01)	0.22 (0.01)	**0.00**	0.01 (0.14)	0.00 (0.05)	0.95
N	13,053			2,022		
Model fit	χ^2^ = 100.635, *df* = 10, *p* < 0.001, CFI = 0.978, RMSEA = 0.026, SRMR = 0.013			χ^2^ = 11.133, *df* = 10, *p* = 0.35, CFI = 0.996, RMSEA = 0.007, SRMR = 0.016		

*These estimates include control variables. P-values ≤ 0.05 are presented in bold.*

**TABLE 4:** Total and indirect effects to children’s behavioral difficulties.

	MCS			NEPS		
Predictor(s)	*B* (*SE*)	β (*SE*)	*p*	*B* (*SE*)	β (*SE*)	*p*
**Parental education**						
Total effect	–0.09 (0.01)	–0.16 (0.01)	**0.00**	–0.17 (0.02)	–0.25 (0.03)	**0.00**
Via sensitive parent–child interactions	–0.00 (0.00)	–0.01 (0.00)	**0.00**	–0.01 (0.01)	–0.01 (0.01)	**0.05**
Via vocabulary skills	–0.01 (0.07)	–0.02 (0.00)	**0.00**	–0.01 (0.01)	–0.02 (0.01)	**0.04**
Via sensitive parent–child interactions and vocabulary skills	–0.00 (0.00)	–0.00 (0.00)	**0.00**	0.00 (0.00)	0.00 (0.00)	0.95
Via negative disciplinary practices	0.00 (0.00)	0.00 (0.00)	0.86	–0.01 (0.00)	–0.01 (0.01)	0.07
**Parental psychological distress**						
Total effect	0.50 (0.03)	0.18 (0.01)	**0.00**	0.09 (0.03)	0.12 (0.04)	**0.00**
Via sensitive parent–child interactions	0.07 (0.00)	0.00 (0.00)	**0.00**	0.00 (0.00)	–0.00 (0.00)	0.88
Via negative disciplinary practices	0.07 (0.01)	0.02 (0.00)	**0.00**	0.01 (0.01)	0.01 (0.01)	0.06
Via sensitive parent–child interactions and vocabulary skills	0.00 (0.00)	0.00 (0.00)	**0.00**	0.00 (0.00)	0.00 (0.00)	0.96
**Difficult temperament (Child)**						
Total effect	0.05 (0.01)	0.07 (0.01)	**0.00**	0.03 (0.01)	0.08 (0.04)	**0.02**
Via negative disciplinary practices	0.02 (0.00)	0.03 (0.00)	**0.00**	0.00 (0.00)	0.00 (0.00)	0.07

*P-values ≤ 0.05 are presented in bold.*

Accounting for child’s sex, formal child care attendance under 36 months, and family net equivalent income, sensitive interactions and children’s vocabulary skills were associated significantly with lower behavioral difficulties in both countries: Children who experienced a higher level of sensitive parent–child interactions and/or had advanced vocabulary skills in toddlerhood showed comparatively fewer behavioral difficulties at age 5. Both sensitive parental interactions and children’s vocabulary skills partially mediated the effect of parental education levels on behavioral difficulties (MCS: β = –0.01 and –0.02, *p*s < 0.001; NEPS: β = –0.01 and –0.02, *p*s < 0.05). In other words, children with higher educated parents were more likely to acquire advanced vocabulary skills or to receive higher levels of parental sensitive interactions that might protect them from developing later behavioral difficulties. In addition, sensitive interactions were associated significantly with children’s vocabulary skills in the United Kingdom, but not in Germany.

At the same time, parental distress, children’s difficult temperament, and parents’ negative disciplinary behavior had direct effects on children’s behavioral difficulties at age 5. Children with a higher level of difficult temperament, whose parents suffered from higher levels of psychological distress in the child’s first year of life, or who experienced negative discipline from their parents at age 3 or 4, were more likely to exhibit comparatively higher levels of behavioral difficulties at age 5. Furthermore, negative disciplinary practices partially mediated the effects of parental distress and children’s difficult temperament on children’s behavioral difficulties in the United Kingdom (β = 0.02 and 0.03, *p*s < 0.001). These mediating effects were not significant in Germany (β = 0.01, *p* = 0.06 and β = 0.00, *p* = 0.07).

Taken together, predictor and mediator variables explained about 22% in the United Kingdom and 15% in Germany of the total variance in behavioral difficulties.

### Robustness Check

When models were calculated separately for protective and risk pathways, the associations between the studied variables were almost the same as documented in the main models. The overall pattern of results was similar across the two cohorts. For details, see the Supplementary Figures 2–5.

## Discussion

The current study examined model specifying protective and risk pathways linked to 5-year-olds’ behavioral difficulties within an integrated framework. Drawing on two large national samples from different Western European countries, we found clear support for applying the theoretical perspectives under study to understand the mediating pathways to behavioral difficulties in early childhood. Primarily, findings suggest that both protective and risk pathways simultaneously influence children’s behavioral difficulties. In other words, even when accounting for family net equivalent income, the child’s sex, and formal child care attendance under 36 months, higher parental education still has direct and indirect effects through protective pathways (i.e., sensitive parent–child interactions, advanced vocabulary skills) on children’s behavioral difficulties leading to comparatively lower levels; whereas parental psychological distress and children’s difficult temperament have effects on children’s behavioral difficulties directly and indirectly through the risk pathway (i.e., parents’ negative disciplinary practices) associated with comparatively higher difficulties. Second, findings confirm that children’s early vocabulary skills are a subsequent protective mechanism—of sensitive parent–child interactions or of higher parental education in the United Kingdom and only of higher parental education in Germany—that impact on behavioral difficulties. Apart from the aforementioned differences, we found broadly consistent patterns of results across both countries. This indicates that these microlevel mechanisms are generalizable within these Western European countries despite contrasting welfare systems at the time of data collection (United Kingdom: 2000–2001; Germany: 2012–2013) as well as across slightly different operationalizations. Finally, the robustness check indicates comparative results between the integrated model (of both protective and risk pathways) and separated models, and thus hints to a rather independent impact of each pathways.

The findings regarding protective pathways are in line with previous research and demonstrate that children with higher compared to lower educated parents exhibit comparatively fewer behavioral difficulties by either experiencing early sensitive interactions and/or by acquiring more advanced vocabulary skills (e.g., Weinert et al., 2017; Rose et al., 2018). In particular, these findings are in accordance with prior study using the same German sample (Huang et al., forthcoming). Drawing on the theoretical concepts of sensitive parenting behaviors (Vygotsky, 1978; Ainsworth, 1979), this evidence indicates that children who experience sensitive interactions and/or acquire advanced vocabulary skills in early childhood tend to show fewer behavioral difficulties at age 5. Similarly, the findings regarding risk pathways support the previous evidence drawing on FSM (e.g., Bates et al., 2014; Gard et al., 2020) and illustrate the risk pathway (i.e., negative discipline) as a link between parental distress and/or children’s difficult temperament and children’s behavioral difficulties.

However, unlike recent evidence from Bornstein et al. (2020), our hypothesized association between sensitive parent–child interactions and children’s vocabulary skills does not emerge in Germany. This could be due to the fact that not all aspects of parenting behaviors affect children’s vocabulary skills (Huang et al., forthcoming). Thus, different aspects of parenting behavior may exert differential impacts on children’s domain-specific development (e.g., Bornstein et al., 2008). Although Bornstein et al. (2020) found a significant association between their globally assessed maternal sensitivity and children’s language skills, they emphasized that this association may be accounted for the verbal interactions measured in the maternal sensitivity. Furthermore, sensitive parent–child interactions (particularly verbal stimulation) might also be associated more strongly with children’s expressive vocabulary but less with receptive vocabulary, because even parent–child interactions in book-reading situations reveal stronger effects on expressive skills, but a weaker or no effect on children’s receptive vocabulary (e.g., DeTemple, 2001; Bojczyk et al., 2016).

Moreover, it is worth noting that in both date sets, the results from robustness check also hold when protective and risk pathways are modeled separately. These findings differ from those of Totsika et al. (2019)—in which the effects of protective pathways disappeared once the risk pathway was simultaneously added to the models. Thus, contrary to Totsika et al. (2019), our analyses suggest that the effects of protective pathways (i.e., sensitive interactions and advanced vocabulary skills) on typically developing children’s behavioral difficulties are not simply markers for the absence of risk factors; and the impact of risk pathways (i.e., negative discipline) is not attenuated when protective factors are considered. Their effects on behavioral difficulties are independent and robust. This difference to Totsika et al.’s (2019) study might be associated with the age differences between the samples (i.e., behavioral outcomes at age 7), because sensitive parenting behavior might be more predictive for younger children (Gardner et al., 2003). Furthermore, the discrepancy could also be that Totsika et al. (2019) used sample in risk circumstance (e.g., children with intellectual disability, experience of poverty), whereas we used typically developing samples. Nonetheless, previous research has claimed that the substantial effects of risk factors might disappear when protective factors are controlled, because the latter appear to influence both risk factors and children’s behavioral difficulties (e.g., Haggerty et al., 1996). However, the protective and risk factors in our both data sets were not interrelated. This suggests that parents’ sensitive interactions are not associated with parental disciplinary strategies. Both factors reflect different dimensions of parenting behaviors. Once again, evidence here emphasizes the multidimensional nature of parenting behaviors—sensitive parent–child interactions and parental disciplinary strategies are different dimensions (Bornstein et al., 2008; Huang et al., forthcoming).

Finally, although our analysis included important indicators of parental education, parenting behavior, and other explanatory factors (e.g., parents’ and children’s characteristics), our models explained only 22 and 15% of the variance in 5-year-olds’ behavioral difficulties in the United Kingdom and Germany. Hence, other factors must account for the unexplained outcome variance. For example, sensitive parent–child interactions in our study were observed directly during interviews or coded from a 10-min videotaped play situation. The frequency of these interactions in daily life might better help to determine the mechanisms. Furthermore, although we focus on the impact of family, we also recognize the importance of extrafamilial childcare—by controlling for formal childcare attendance under 36 months. In both countries, nearly all children began Kindergarten at age 3 (and thus 2 years before outcome assessment). Relations with peers and caregivers within the Kindergarten environment might also contribute to children’s later behavioral outcomes. The differences in explained variance between the two countries might be due to the different welfare systems and the different measurements in the two data sets. However, the notable finding here is the broadly similar mechanisms despite these differences between countries.

### Strengths and Limitations

This study contributes to the extensive research on mediational pathways linked to children’s behavioral difficulties by tracing 5-year-olds’ behavioral difficulties in a range of novel ways: First, it advances our understanding of protective mechanisms by examining sensitive parent–child interactions as protective pathways linking parental education and children’s behavioral difficulties. In particular, drawing on theoretical and empirical research, we also included children’s vocabulary skills as a subsequent mechanism in our models. Findings indicate that alongside sensitive interactions (attachment theory), language skills (language stimulating scaffolding behavior) serve as an important means of communication to facilitate behavioral development in early childhood. As a second strength, unlike previous studies, we simultaneously considered stressors from both parents’ and children’s perspectives indirectly *via* the effect of negative discipline on behavioral difficulties. Third, by using two large national samples from different countries, this study delivers generalizable evidence. These large-scale data sets also allow us to control for the known effects of child and parent covariates. Measurements were theoretically well-designed and based on observation, parent questionnaires, and vocabulary tests with empirically substantiated fit characteristics. Most notably, we identified the independent effects of protective and risk pathways. Fourth, this generalizable evidence suggests that specific preventive programs to help parents deliver more sensitive interactions and efficient disciplinary strategies to their children could prevent problematic child behavior. Furthermore, programs additionally promoting children’s vocabulary skills could also, in turn, help prevent children from exhibiting behavioral difficulties.

That said, this study is not without limitations. First, results cannot be interpreted causally, because structural equation modeling draws on correlational evidence and does not experimentally examine causal effects among these factors. Second, the Cronbach’s alphas of some measures are not very high (α: 0.51–0.80). Given that the assumptions (e.g., tau equivalent) of Cronbach’s alpha are almost never fulfilled, we additionally calculated the composite reliabilities (CRs) of the constructs (which were specified as latent variables) and found that the CRs were not very high either (see Supplementary Table 2). The comparably low internal consistencies of some measures (e.g., temperament) might result from the fact that children were very young (7 or 9 months old); characteristics of children at such a young age might not be as coherent or consistent as in older children (Petersen et al., 2015). Furthermore, due to time constraints in large-scale studies the instruments had to be rather short. Scales including more items might be more reliable (Streiner, 2003) and warranted in further research. Related to this issue, we additionally ran two sensitivity tests including the measurement errors to check whether our results are robust to the low reliabilities of some measures. We applied single-indicator latent variables for item parcels with respect to parental distress, child temperament, and negative disciplinary practices in one model and included the full measurement models in another (replacing the item parcels). Overall, we found very similar estimates in both tests compared to our original models (for details see Supplementary Tables 3, 4). Third, although we investigated the impact of two different forms of parenting behavior—observed parent–child interactions and self-reported disciplinary parenting behavior—on children’s behavioral difficulties, only 1% (MCS) and 3% (NEPS) of the parents included in the studies are fathers. Hence, we can hardly draw conclusions on the influence of fathers. Given the increasingly acknowledged importance of the father’s role in parenting behavior, future studies should further examine their role in these mechanisms. Fourth, children’s behavioral difficulties are parent-reported. This might be of limited accuracy due to social desirability and the variation in parental observation. Although we had valid measures of the three-factor behavioral difficulties, future studies using multi informant assessment (e.g., teacher-report) are needed.

## Conclusion

In early childhood, sensitive parent–child interactions or possessing advanced vocabulary skills in toddlerhood can protect children from exhibiting behavioral difficulties (i.e., protective pathways). In contrast, experiencing negative discipline may elicit behavioral difficulties (i.e., risk pathways). These two pathways proved independent in our study. This emphasizes that even for children from risk groups (i.e., from families with lower parental education, who have difficult temperament, or have parents suffering from psychological distress), programs that enhance early parent–child interactions and children’s vocabulary skills or support parents in promoting appropriate disciplinary strategies hold considerable potential for preventing early behavioral difficulties.

## Data Availability Statement

Publicly available datasets were analyzed in this study. This data can be found here: http://ukdataservice.ac.uk, https://www.neps-data.de/Data-Center/Data-and-Documentation/Starting-Cohort-Newborns.

## Ethics Statement

Ethical review and approval was not required for the study on human participants in accordance with the local legislation and institutional requirements. Written informed consent to participate in this study was provided by the participants’ legal guardian/next of kin.

## Author Contributions

WH designed the research questions as well as the computational modeling, calculated the analyses and drafted the manuscript. All authors contributed to the specification of the hypotheses, the exact modeling and revision of the manuscript.

## Conflict of Interest

The authors declare that the research was conducted in the absence of any commercial or financial relationships that could be construed as a potential conflict of interest.

## Publisher’s Note

All claims expressed in this article are solely those of the authors and do not necessarily represent those of their affiliated organizations, or those of the publisher, the editors and the reviewers. Any product that may be evaluated in this article, or claim that may be made by its manufacturer, is not guaranteed or endorsed by the publisher.

## Footnotes

1
https://cls.ucl.ac.uk/wp-content/uploads/2017/07/MCS2-CAPI-Questionnaire-Child-Measurements.pdf

## Dedication

This paper is dedicated to our co-author James Law who passed away too early in October 2021.
