# Supplementary material for: The Emergence of 5-Year-Olds’ Behavioral Difficulties: Analyzing Risk and Protective Pathways in the United Kingdom and Germany
Source: Front Psychol. 2022 Jan 5;12:769057. doi: 10.3389/fpsyg.2021.769057 (PMC8767965; doi:10.3389/fpsyg.2021.769057)
Supplement: Supplementary file 1 [file Data_Sheet_1.docx]

**Supplementary Material**

**Table 1.** UK qualifications and CASMIN categories.

| Reduced categories | CASMIN full code and description | UK qualifications |
| --- | --- | --- |
| Low | 1a  Inadequately completed general elementary education | None of these qualifications (this excludes any overseas qualifications) No qualifications |
|  | 1b  Inadequately completed general elementary education | GCSE grades D–G (academic) |
|  | 1c  Basic vocational qualification or general elementary education and basic vocational qualification | NVQ SVQ GSVQ level 1 (vocational) |
|  | 2a  Intermediate vocational qualification or intermediate general education plus basic vocational qualification | NVQ SVQ GSVQ level 2 (vocational) |
| Middle | 2b  Intermediate general qualification | O level GCSE grade A–C (academic) |
|  | 2c (Vocational)  Full general maturity certificate (vocational) | NVQ SVQ GSVQ level 3 (vocational) |
|  | 2c (General)  Full general maturity certificate (academic) | A AS S Levels (academic) |
| High | 3a  Lower tertiary certificate | Diplomas in higher education, nursing, or other medical qualifications, NVQ level 4 |
|  | 3b  Higher tertiary certificate | First degree, higher degree, professional qualifications at degree level, NVQ level 5 |

**Table 2**. Reliabilities of investigated constructs.

|  | MCS | | | NEPS | | |
| --- | --- | --- | --- | --- | --- | --- |
|  | Items | Cronbach’s alpha | Composite reliability | Items | Cronbach’s alpha | Composite reliability |
| Parental psychological distress | 9 | .72 | .74 | 3 | .65 | .67 |
| Difficult temperament (child) | 3 | .54 | .55 | 3 | .51 | .54 |
| Negative disciplinary practices | 6 | .70 | .65 | 4 | .67 | .73 |

**Table 3.** Results from models using single-indicator latent variables for MCS and NEPS.

|  | MCS | | |  | NEPS | | |
| --- | --- | --- | --- | --- | --- | --- | --- |
|  | *B (SE)* | *ß (SE)* | *p* |  | *B (SE)* | *ß (SE)* | *p* |
| **Behavioral difficulties** 🡪 |  |  |  |  |  |  |  |
| Parental education | −.08 (.01) | −.13 (.01) | .00 |  | −.14 (.03) | −.21 (.04) | .00 |
| Parental psychological distress | .55 (.04) | .17 (.01) | .00 |  | .10 (.05) | .10 (.06) | .04 |
| Difficult temperament (child) | .02 (.01) | .02 (.02) | .12 |  | .04 (.03) | .04 (.06) | .12 |
| Negative disciplinary practices | .27 (.01) | .27 (.01) | .00 |  | .07 (.04) | .08 (.05) | .10 |
| Sensitive parent–child interactions | −.03 (.01) | −.06 (.01) | .00 |  | −.09 (.05) | −.07 (.04) | .09 |
| Vocabulary skills | −.07 (.01) | −.14 (.01) | .00 |  | −.04 (.02) | −.08 (.04) | .03 |
| **Negative disciplinary practices** 🡪 |  |  |  |  |  |  |  |
| Parental education | −.01 (.01) | −.01 (.01) | .54 |  | −.13 (.05) | −.14 (.05) | .01 |
| Parental psychological distress | .41 (.07) | .11 (.02) | .00 |  | .23 (.05) | .16 (.04) | .02 |
| Difficult temperament (child) | .21 (.02) | .23 (.02) | .00 |  | .13 (.05) | .23 (.08) | .01 |
| **Sensitive parent–child interactions** 🡪 |  |  |  |  |  |  |  |
| Parental education | .13 (.01) | .11 (.01) | .00 |  | .08 (.03) | .15 (.05) | .01 |
| Parental psychological distress | −.34 (.10) | −.05 (.01) | .00 |  | .00 (.03) | .01 (.04) | .89 |
| **Vocabulary skills** 🡪 |  |  |  |  |  |  |  |
| Parental education | .13 (.01) | .12 (.01) | .00 |  | .31 (.07) | .20 (.05) | .00 |
| Sensitive parent–child interactions | .20 (.01) | .22 (.01) | .00 |  | .05 (.13) | .02 (.05) | .69 |
| N | 13,053 | | |  | 2,022 | | |
| Model fit | χ^2^ = 91.410, *df* = 10, *p* < .001, CFI = .985, RMSEA = .025, SRMR = .012 | | |  | χ^2^ = 11.167, *df* = 10, *p* = .345, CFI = .998, RMSEA = .008, SRMR = .016 | | |

*Note*. These estimates include control variables.

Parental psychological distress, difficult temperament (child), and negative disciplinary practices were modelled as single-indicator latent variables to represent respective construct with each latent variable being measured by its corresponding scale score (parcel). Each single-indicator latent variable was specified by fixing the scale score’s factor loading to one and fixing its error variance (δ_x_) to one minus the scale’s reliability coefficient multiplied by the variance of the composite-score (δ_x_ = (1 – ρ)*VAR(X)).

**Table 4.** Results from the full measurement models for MCS and NEPS.

|  | MCS | | |  | NEPS | | |
| --- | --- | --- | --- | --- | --- | --- | --- |
|  | *B (SE)* | *ß (SE)* | *p* |  | *B (SE)* | *ß (SE)* | *p* |
| **Behavioral difficulties** 🡪 |  |  |  |  |  |  |  |
| Parental education | −.07 (.01) | −.15 (.01) | .00 |  | −.16 (.03) | −.27 (.04) | .00 |
| Parental psychological distress | .43 (.04) | .20 (.02) | .00 |  | .09 (.06) | .13 (.07) | .09 |
| Difficult temperament (child) | .03 (.01) | .05 (.02) | .01 |  | .06 (.03) | .11 (.07) | .10 |
| Negative disciplinary practices | .22 (.01) | .29 (.02) | .00 |  | .08 (.05) | .13 (.07) | .10 |
| Sensitive parent–child interactions | −.03 (.01) | −.08 (.02) | .00 |  | −.09 (.06) | −.09 (.06) | .13 |
| Vocabulary skills | −.07 (.01) | −.16 (.01) | .00 |  | −.04 (.02) | −.10 (.05) | .04 |
| **Negative disciplinary practices** 🡪 |  |  |  |  |  |  |  |
| Parental education | −.01 (.01) | −.02 (.02) | .24 |  | −.15 (.03) | −.14 (.05) | .00 |
| Parental psychological distress | .28 (.05) | .10 (.02) | .00 |  | .23 (.08) | .19 (.06) | .00 |
| Difficult temperament (child) | .19 (.02) | .22 (.02) | .00 |  | .16 (.06) | .19 (.07) | .01 |
| **Sensitive parent–child interactions** 🡪 |  |  |  |  |  |  |  |
| Parental education | .13 (.01) | .11 (.01) | .00 |  | .08 (.03) | .15 (.05) | .01 |
| Parental psychological distress | −.30 (.08) | −.06 (.01) | .00 |  | .01 (.04) | .02 (.05) | .72 |
| **Vocabulary skills** 🡪 |  |  |  |  |  |  |  |
| Parental education | .13 (.01) | .12 (.01) | .00 |  | .31 (.07) | .20 (.05) | .00 |
| Sensitive parent–child interactions | .20 (.01) | .22 (.01) | .00 |  | .05 (.14) | .02 (.05) | .67 |
| N | 13,053 | | |  | 2,022 | | |
| Model fit | χ^2^ = 6827.342, *df* = 708, *p* < .001, CFI = .869, RMSEA = .026, SRMR = .031 | | |  | χ^2^ = 682.179, *df* = 401, *p* < .001, CFI = .905, RMSEA = .019, SRMR = .039 | | |

*Note*. These estimates include control variables.

The full measurement models of children’s behavioral difficulties, parental psychological distress, difficult temperament (child), and negative disciplinary practices were included in the structural model. Given the multidimensionality of the first construct and the unidimensionality of the latter three constructs, we applied second-order (containing three factors) and first-order models, respectively.

**Figure 1.** Working models for examining associations in the current study.


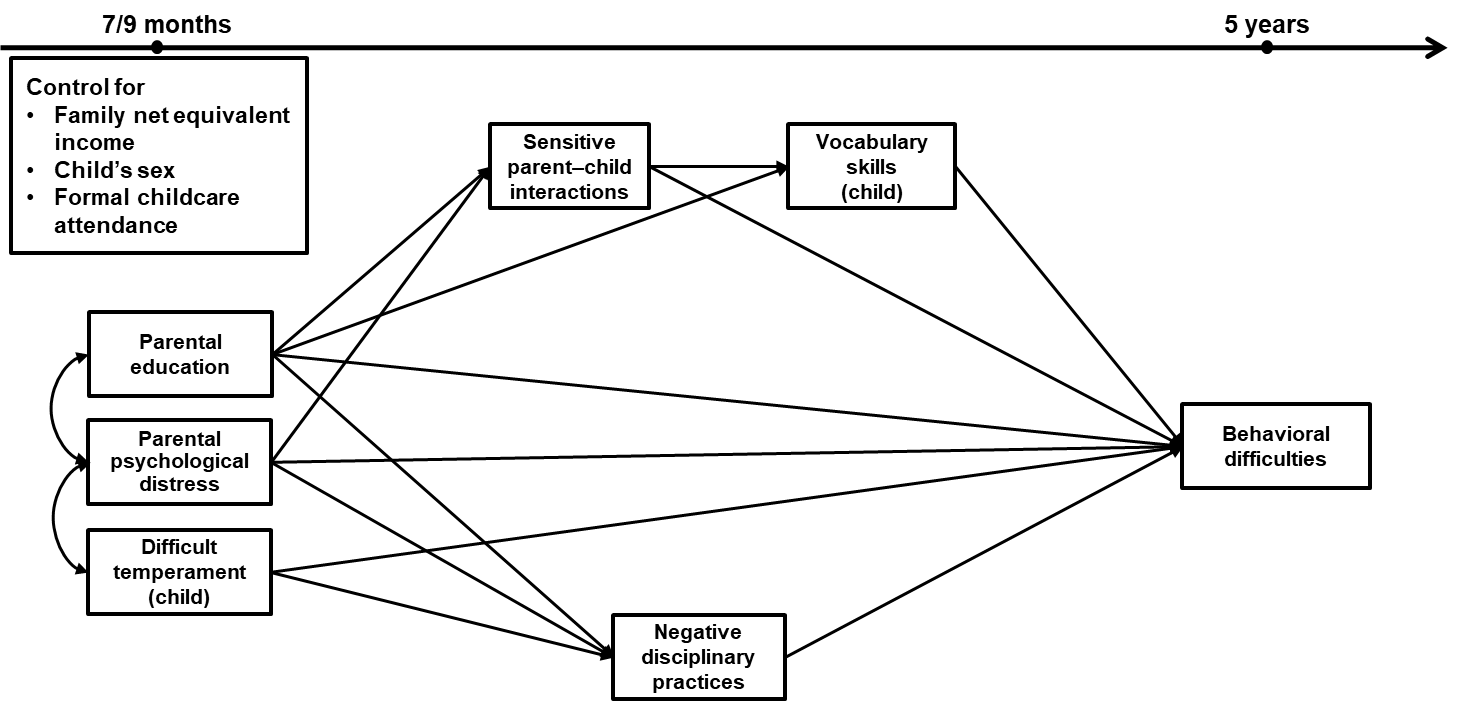


**Figure 2.** Standardized estimates of the conditional Model A1 using MCS in UK.


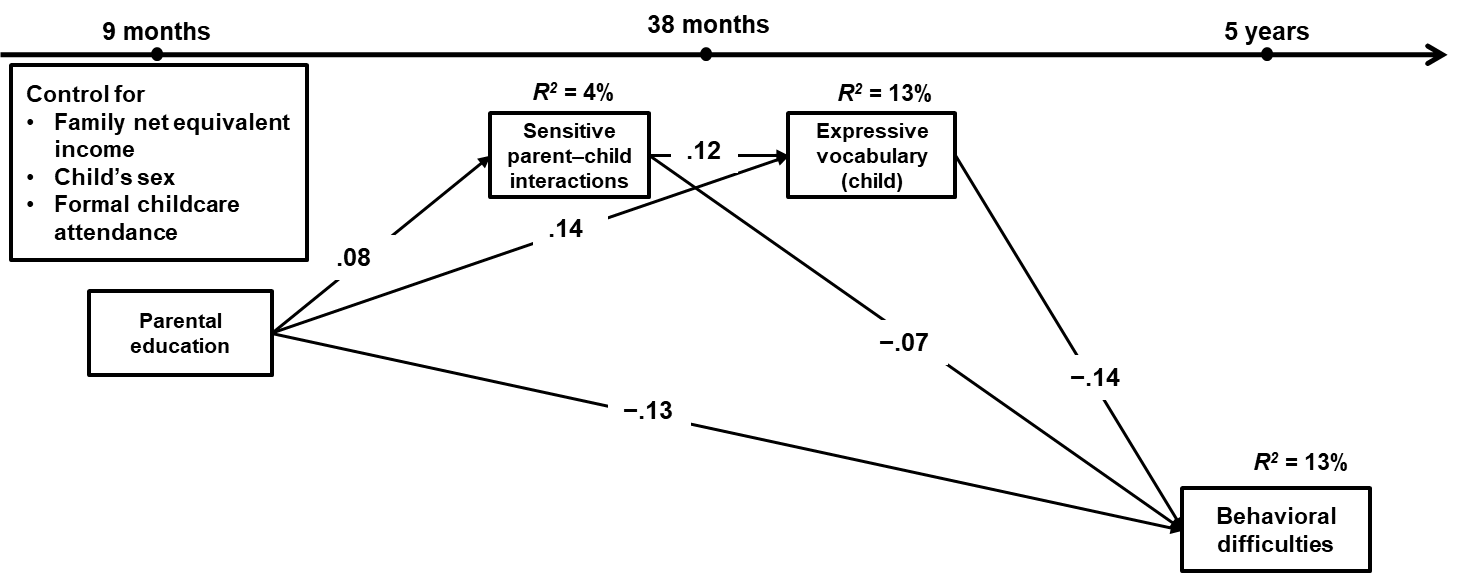


*Note.* All coefficients are significant at the *p* < .05 level. *N* = 13,053, χ^2^ = 11.083, *df* = 5, *p* = .050, CFI = .998, RMSEA = .010, SRMR = .006.

**Figure S3.** Standardized estimates of the conditional Model A1 using NEPS in Germany.

**
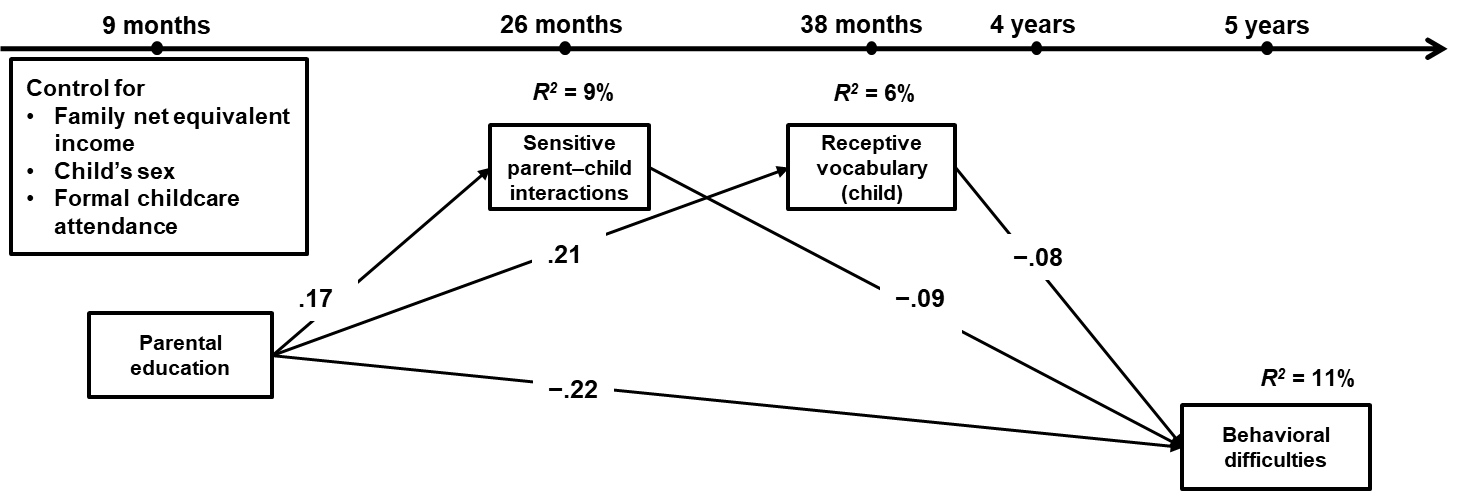
**

*Note.* All coefficients are significant at the *p* < .05 level. *N* = 2,022, χ^2^ = 7.292, *df* = 5, *p* = .200, CFI = .987, RMSEA = .015, SRMR = .015.

**Figure 4.** Standardized estimates of the conditional Model A2 using MCS in UK.


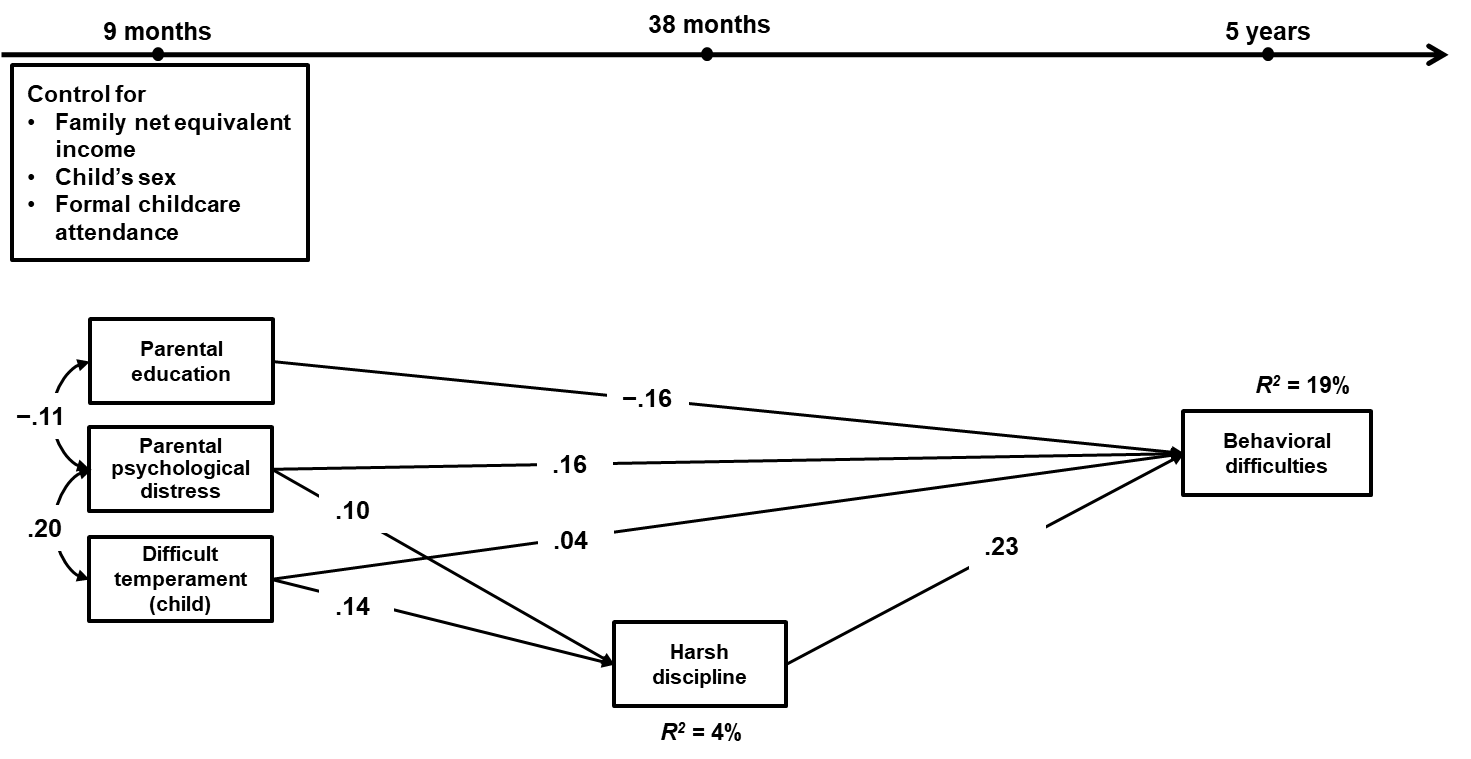


*Note.* All coefficients are significant at the *p* < .05 level. *N* = 13,053, χ^2^ = 79.203, *df* = 3, *p* < .001, CFI = .967, RMSEA = .044, SRMR = .014.

**Figure 5.** Standardized estimates of the conditional Model A2 using NEPS in Germany.


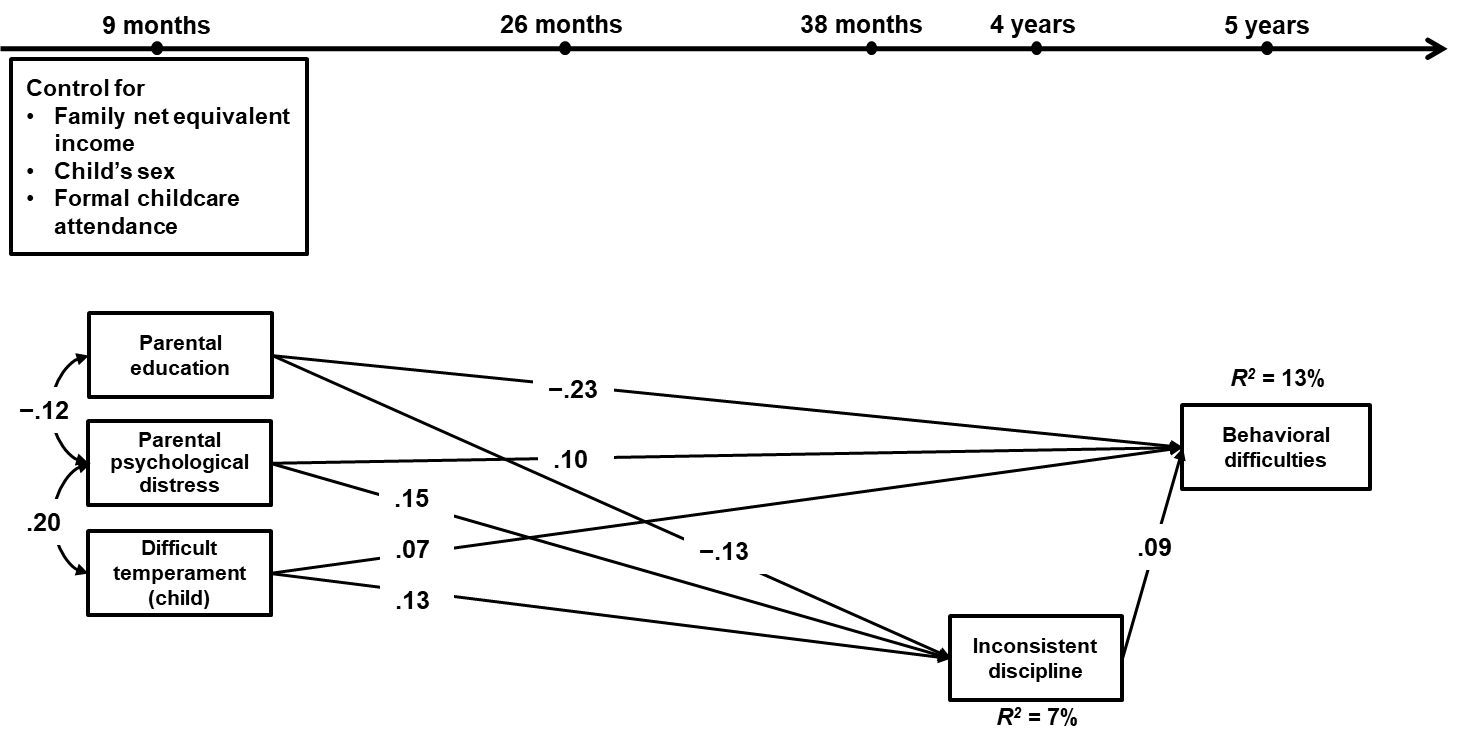


*Note.* All coefficients are significant at the *p* < .05 level. *N* = 2,022, χ^2^ = 5.388, *df* = 3, *p* = .146, CFI = .982, RMSEA = .020, SRMR = .016.
